# Treatment of neck fractures of the fifth metacarpal with MIROS (minimally invasive reduction and osteosynthesis system): a retrospective case-series

**DOI:** 10.1007/s00590-025-04642-5

**Published:** 2025-12-26

**Authors:** Andrea Vescio, Alessandro Barone, Michele Mercurio, Alessia Giofrè, Livio Perticone, Daria Riccelli, Giuseppe Tedesco, Erminia Cofano, Giorgio Gasparini, Filippo Familiari

**Affiliations:** 1 Department of Life Science, Health, and Health Professions, Link Campus University, Rome, Italy; 2 Azienda Ospedaliero Universitario Mater Domini, Catanzaro, Italy; 3 Department of Orthopaedic and Trauma Surgery, Magna Graecia University, Catanzaro, Italy; 4 Azienda Ospedaliera Pugliese Ciaccio, Catanzaro, Italy

**Keywords:** Boxer’s fracture, Fifth metacarpal, MIROS system, Minimally invasive, K-wire fixation

## Abstract

**Background:**

Neck fractures of the fifth metacarpal are frequent hand injuries. Surgical intervention is indicated for fractures with > 30° flexion angulation, rotational deformities, or shortening > 3 mm. Various techniques, including K-wire fixation, plating, and minimally invasive systems can be used. This study evaluates the efficacy of the Minimally Invasive Reduction and Osteosynthesis System (MIROS) for treating displaced fifth metacarpal neck fractures.

**Methods:**

A retrospective review of patients treated with MIROS between 2019 and 2024 was conducted. Patients aged 18–80 years with unstable neck fractures of the fifth metacarpal were included, excluding open, pathological, or polytrauma cases. Radiological outcomes were assessed through Palmar Tilt Angle (PTA) and Lateral Tilt Angle (LTA) measurements pre- and postoperatively. Secondary outcomes included surgery duration, radiation exposure, healing time, and complications.

**Results:**

Fifty-two patients (44 men) were included, with a mean age of 35.1 ± 15.1 years. Postoperative improvements in PTA (from 35.4 ± 16.6 to 15.9° ± 9.3; *p* < 0.0001) and LTA (from 56.7 ± 14.7 to 29.3 ± 11.9; *p* < 0.0001) were significant. Mean surgery duration was 37.4 ± 18.1 min, and radiological healing time averaged 2.3 ± 1.4 months. The complication rate was 7.7%, with three cases of K-wire infection and one case of finger overlap.

**Conclusion:**

MIROS demonstrated effective anatomical restoration, and a low complication rate, proving to be a reliable surgical technique for fifth metacarpal neck fractures. Further prospective, multicenter trials with longer follow-up are necessary to validate these findings.

## Introduction

Neck fractures of the fifth metacarpal, commonly referred to as “boxer’s fractures,” are among the most frequent hand injuries and account for approximately 10% of all hand fractures [1]. These injuries typically occur in young, active individuals or manual laborers and may lead to both cosmetic and, more importantly, functional sequelae, including impairments in metacarpophalangeal joint extension [2, 3]. Management depends on whether the fracture is open or closed and on specific fracture characteristics, such as the degree of angulation, shortening, rotational deformity, and the presence of associated injuries [4–6]. Conservative treatment is appropriate when three criteria are simultaneously satisfied: volar angulation of the metacarpal head less than 30°, absence of rotational deformity, and metacarpal head shortening less than 3 mm. Failure to meet these criteria warrants surgical intervention [7, 8].

Multiple operative techniques have been described, including percutaneous pinning, external fixation, intramedullary devices, combinations of these methods, and open reduction with internal fixation (ORIF) using plates and screws, each with distinct advantages and limitations [7, 9–11].

The concept of minimally invasive surgery [10–13] represents a relatively recent advancement. Contemporary research increasingly emphasizes techniques that replace traditional open approaches with less invasive or percutaneous methods. These approaches aim to minimize soft-tissue disruption and bone sacrifice, thereby facilitating faster functional recovery and improving clinical outcomes [10, 11, 13–15]. This philosophy, often referred to as “Tissue-Sparing Surgery” [3], has shown promising results in promoting rapid healing and reducing postoperative complications [11]. The MIROS (Minimally Invasive Reduction and Osteosynthesis System) [12, 13, 16], composed of isoelastic metal wires coupled with an external fixation device, represents a valuable option within this treatment paradigm. The system is designed to adapt to the morphology of any anatomical region, regardless of size, harnessing the advantages of both intramedullary fixation and external stabilization. It also enables the creation of novel configurations, such as the “Delta” construct used in supracondylar fractures of the femur, humerus, and tibial pilon. However, evidence regarding the use of the MIROS system specifically for fifth metacarpal neck fractures remains limited [13].

Currently, no consensus exists on the optimal treatment strategy for metacarpal neck fractures. The aim of the present study was to evaluate the radiographic outcomes of displaced fifth metacarpal neck fractures treated with the bouquet MIROS system. We hypothesized that MIROS would provide satisfactory results by enabling anatomic restoration of unstable fractures while allowing early mobilization of the injured hand [13].

## Methods

### Study design and sample

A retrospective review of medical records was conducted at the Azienda Ospedaliero-Universitaria “Renato Dulbecco” for patients treated between January 2019 and December 2024. All individuals were admitted through the emergency department. Demographic and clinical variables collected included sex, age at the time of injury, mechanism of trauma, laterality, presence or absence of neurovascular compromise, and whether the fracture was open or closed.

The inclusion criteria comprised patients aged 18–80 years with simple transverse fractures, significant angular deformity, rotational malalignment, or unstable fractures following reduction of a fifth metacarpal neck fracture (AO/OTA classification 77.5.3 A), who underwent surgical treatment with the MIROS System and had complete radiographic follow-up. Exclusion criteria included open or pathological fractures, polytrauma, and incomplete or missing clinical or radiographic data.

### Surgical technique

The surgical procedure (MIROS; Technovare Europa Trading, Anagni, Frosinone, Italy) was performed using a standardized kit consisting of K-wires, fixation clips, and manual introducers. The distal tips of the K-wires were pre-bent to approximately 20° using pliers. Patients were positioned supine with the affected arm supported on a radiolucent hand table. Under fluoroscopic guidance, the dorsoulnar aspect of the metacarpal base was selected as the entry point, ensuring avoidance of the carpometacarpal joint surface and protection of the extensor carpi ulnaris tendon insertion. The K-wires were manually advanced into the medullary canal without crossing the fracture site, with the pre-bent tips oriented palmarly.

Preliminary fracture reduction was achieved by flexing both the metacarpophalangeal (MCP) and proximal interphalangeal (PIP) joints to 90°, allowing the metacarpal head to be guided back into anatomical alignment. Once an adequate reduction was confirmed fluoroscopically, the K-wires were advanced across the fracture into the metacarpal head. The bent tips were subsequently rotated dorsally and directed in slightly divergent trajectories—dorsoradial and dorsoulnar.

Following K-wire placement, alignment and rotational correction were verified by assessing digital range of motion. Proximally, the wires were bent and trimmed to approximately 1 cm above the skin surface. Fixation clips were then applied to create a construct analogous to an external fixator, permitting fine adjustment of rotation, length, and axial collapse.

Postoperative management included immediate mobilization without splinting or bandaging, facilitating early functional recovery. Hardware removal was scheduled at 30 days postoperatively following radiographic confirmation of adequate fracture healing.

### Primary outcome: radiological evaluation

**Fig. 1:**
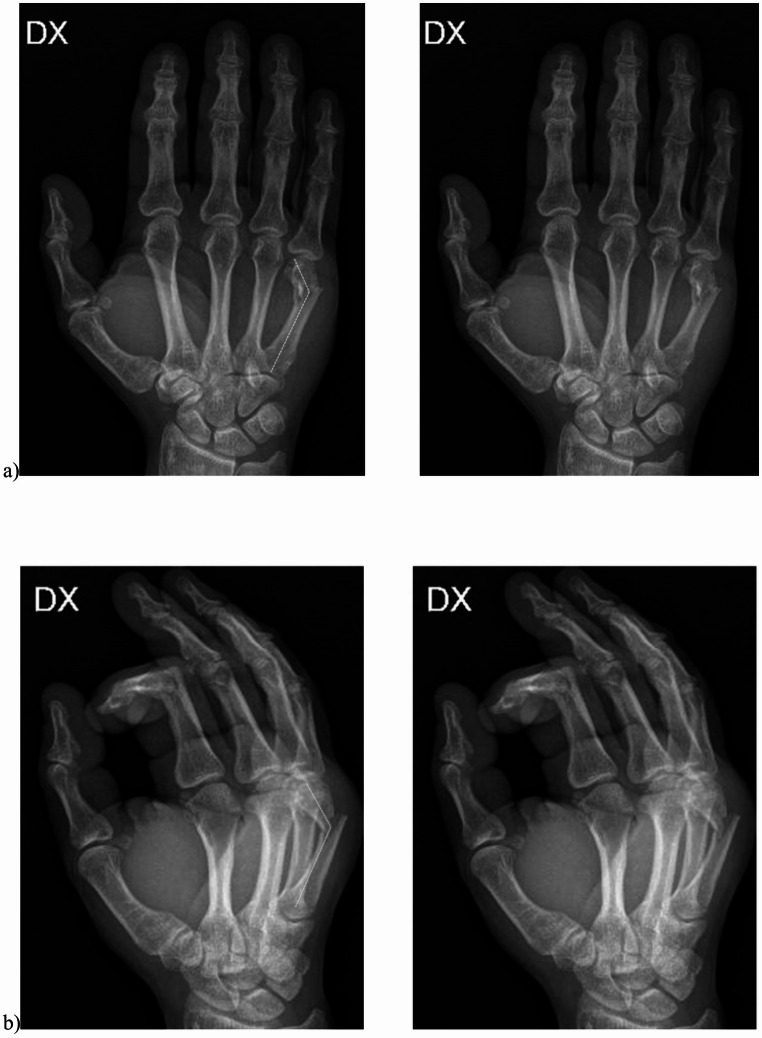
Pre-operatively palmar tilt angle (PTA) (**a**) and lateral tilt angle (LTA) (**b**) assessment

**Fig. 2:**
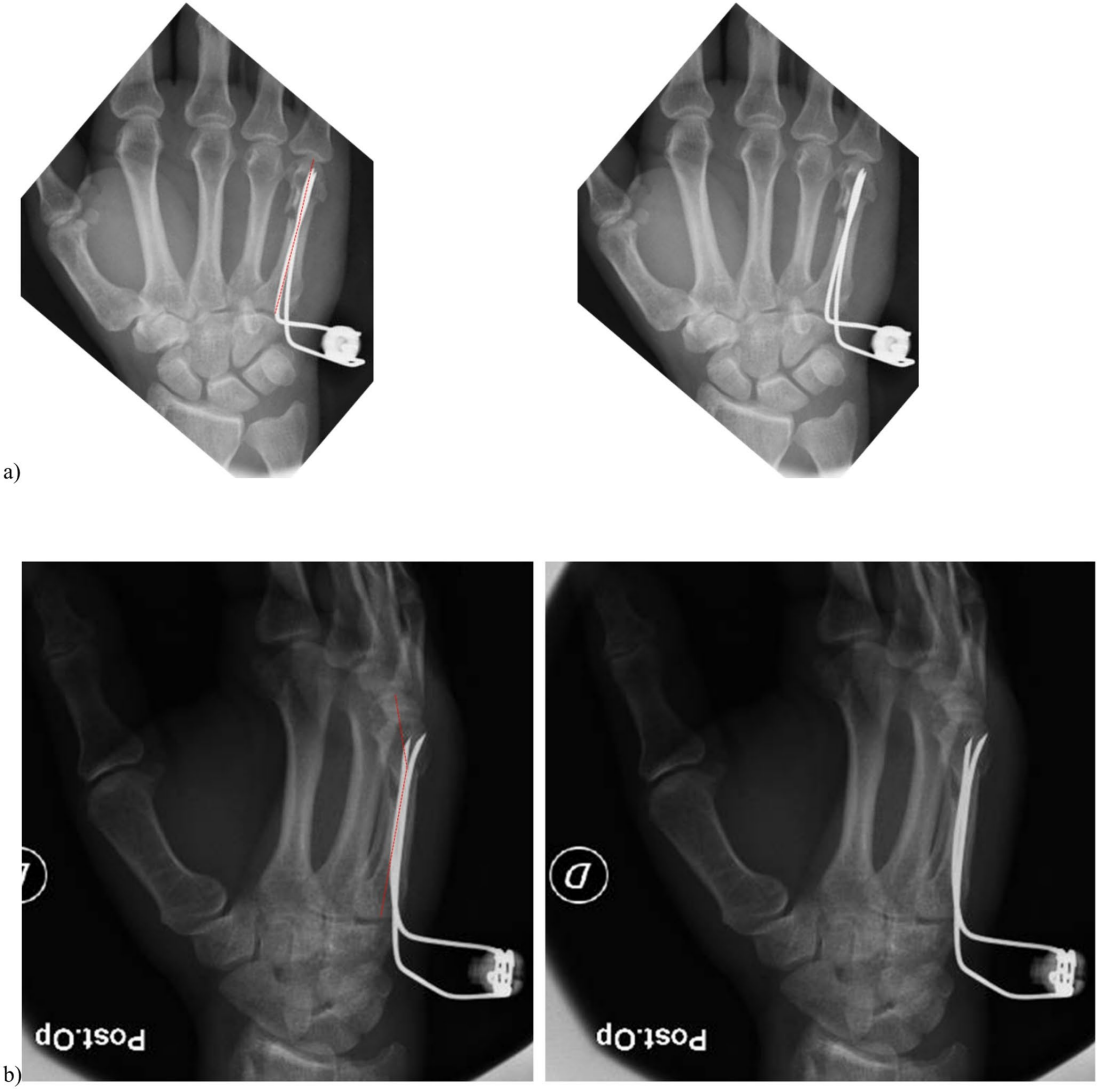
Post-operatively palmar tilt angle (PTA) (**a**) and lateral tilt angle (LTA) (**b**) assessment

The injured hand underwent anteroposterior (AP) and lateral radiographic evaluation to assess the fracture location, configuration, and degree of displacement. Radiographs were obtained at admission, immediately after surgical treatment, and at 4 weeks postoperatively. Preoperative (Fig. 1a and b) and postoperative (Fig. 2a and b) radiographs were analyzed to measure the Palmar Tilt Angle (PTA) [8]—defined as the angle between the longitudinal axis of the metacarpal shaft and the distal articular surface in the sagittal plane—and the Lateral Tilt Angle (LTA) [8], defined as the angle between the distal articular surface and a line perpendicular to the metacarpal shaft in the coronal plane. Two independent, blinded evaluators performed all radiographic measurements using calibrated digital software.

### Secondary outcome

Secondary outcomes included radiation exposure (measured in seconds), surgical duration (measured in minutes), and radiological healing time, defined as the presence of bone bridging, callus formation, or trabeculae across three out of four cortices on two orthogonal radiographic views (measured in months). Moreover, complications were recorded.

### Statistical analysis

Continuous variables were summarized as means ± standard deviations, while categorical variables were expressed as frequencies and percentages. Preoperative and postoperative radiological measurements were compared using paired t-tests, with statistical significance set at *p* < 0.05. All analyses were performed using the 2016 GraphPad Software (GraphPad Inc, USA).

## Results

### Sample characteristics

A total of 156 subjects were included in the study 52 patients (44 men and 8 women) with fractures of the fifth metacarpal neck met the inclusion criteria. The mean age was 35.1 ± 15.1 years. The 75.9% of fractures involved the right hand (Table 1). The traumatic mechanism may result from falling (2 patients, 3.8%), domestic accident (29 patients, 55.8%), crushing injury (3 patients, 5.8%), or contact trauma in sports (18 patients, 34.6%).

### Radiological outcomes

Significant improvements were observed in both PTA and LTA postoperatively. PTA decreased from a mean of 35.4° ± 16.6° preoperatively to 15.9° ± 9.3° postoperatively (*p* < 0.0001). LTA decreased from a mean of 56.7° ± 14.7° preoperatively to 29.3° ± 11.9° postoperatively (*p* < 0.0001) (Table 1).

**Table 1:** Demographic characteristics of patients managed by MIROS system

Sample	Mean age	Injured side (R/L)		Palmar tilt angle pre-op	Palmar tilt angle post-op	Lateral tilt angle pre-op	Lateral tilt angle post-op
		Right	Left				
52	35.1 ± 15.1	41	13	35.4 ± 16.6	15.9° ± 9.3	56.7 ± 14.7	29.3 ± 11.9
				*P* < 0.0001		*P* < 0.0001	

### Secondary outcomes

Radiation exposure was recorded at a mean of 21.7 ± 16.6 s, while the mean surgery duration was 37.4 ± 18.1 min. The average radiological healing time was 2.3 ± 1.4 months. No instances of nonunion were observed. The overall complication rate was 7.7%, comprising three cases of K-wire infection and one case of finger overlap.

## Discussion

The MIROS system demonstrated substantial efficiency and effectiveness in the surgical management of fifth metacarpal neck fractures, achieving consistent restoration of normal alignment in both radiographic planes and a low complication rate. These results add to the growing body of literature supporting MIROS as a viable alternative to traditional surgical techniques.

A wide range of operative strategies for fifth metacarpal neck fractures has been described [15, 17], each with specific advantages and limitations. K-wire fixation remains widely used and encompasses several configurations. In cross-pinning, two bicortical wires are inserted retrogradely without crossing the fracture site, thereby reducing the risk of rotational malalignment. Crucifix pinning combines a larger retrograde intramedullary wire with a thinner transverse wire stabilizing both the injured and adjacent metacarpal. Bouquet pinning involves multiple antegrade K-wires inserted into the medullary canal, with their distal ends bent dorsally to maintain reduction [15]. Lazy-S pinning employs a single antegrade K-wire with opposing curves to enhance stability [6]. Transverse pinning, most commonly used in fourth- and fifth-metacarpal fractures, stabilizes the fracture by inserting K-wires from the ulnar aspect into the adjacent metacarpals [18].

Intramedullary fixation using headless compression screws offers the advantage of buried hardware, eliminating the need for removal. Plate and screw fixation, although traditionally considered biomechanically superior, has been shown in comparative studies to have similar peak load and stiffness to K-wire constructs, calling this presumed superiority into question [15, 17].

A recent network meta-analysis comparing treatments for boxer’s fractures identified meaningful differences across techniques. Retrograde intramedullary pinning was associated with greater early postoperative pain compared to anterograde intramedullary pinning (AIMP). However, AIMP showed higher rates of implant migration and neurologic complications compared with plating, as well as a higher risk of delayed union compared with transverse pinning [15].

Despite their widespread use, percutaneous intramedullary K-wire techniques—whether antegrade or retrograde—carry inherent limitations, including joint transfixation, technical challenges in proximal exposure, the need for splinting or bracing, limited rotational control, and postoperative stiffness. To address these shortcomings, the use of a clamp-based construct integrating multiple K-wires has been proposed. This hybrid configuration creates a more robust fixation, eliminates the need for postoperative immobilization, and facilitates early mobilization while improving control of rotation and length [19].

MIROS leverages these principles by providing an external fixator–like construct with isoelastic wires, minimizing soft-tissue disruption compared with plating and permitting immediate mobilization without splinting [12, 20]. Its design reduces the risk of implant migration and enhances rotational stability. Nonetheless, MIROS shares some of the complications associated with K-wire methods, including pin-site infection and finger overlap—findings consistent with previous reports [20, 21], where infection rates ranged from 4.7% to 21.4%. Gu et al. [22] similarly found no significant difference in complications between modified K-wire fixation and medial locking plates, supporting the safety of minimally invasive fixation strategies. However, the increased use of fluoroscopy intrinsic to MIROS may contribute to higher cumulative radiation exposure.

Radiographically, the present study demonstrated significant improvements in alignment across both assessed planes. The PTA decreased from a mean of 35.4° ± 16.6° preoperatively to 15.9° ± 9.3° postoperatively, while the LTA improved from 56.7° ± 14.7° to 29.3° ± 11.9° (both *p* < 0.0001). These outcomes align with previously published findings. Gu et al. [22] reported correction of deformity from a mean angulation of 40.0° ± 3.7° to 17.6° ± 1.7° with modified K-wire fixation. Similarly, Kim and Kim [23] documented comparable reductions in apex dorsal angulation using both antegrade and retrograde intramedullary techniques.

The mean operative time in the present study (37.4 ± 18.1 min) compares favorably with reported durations. Chen et al. [24] observed shorter operative times for K-wire fixation (mean 27 min) compared with medial locking plates (mean 48 min). Gu et al. [22] reported a mean of 44.8 min. The slightly shorter operative times in the current series may reflect the procedural efficiency associated with the MIROS construct. Radiographic healing occurred at a mean of 2.3 ± 1.4 months, and no cases of nonunion were identified, further supporting the technique’s effectiveness.

To the authors’ knowledge, this is the first study to evaluate the MIROS system specifically for fifth metacarpal neck fractures. The study has several strengths, including a five-year retrospective design capturing diverse real-world presentations, and a standardized surgical protocol ensuring consistent application of the technique. However, limitations include the inherent biases of retrospective research, potential gaps in clinical documentation, lack of a control group, and the absence of long-term clinical and functional outcomes. Future multicenter, prospective studies with extended follow-up are warranted to validate these findings and compare MIROS directly with alternative fixation methods.

In conclusion, the MIROS system achieved effective anatomical restoration with a low complication rate, representing a reliable surgical option for fifth metacarpal neck fractures. Nevertheless, further prospective trials are needed to confirm these results and to establish comparative effectiveness against other fixation techniques.

## Data Availability

No datasets were generated or analysed during the current study.
